# Automated Multi-Lesion Detection for Referable Diabetic Retinopathy in Indigenous Health Care

**DOI:** 10.1371/journal.pone.0127664

**Published:** 2015-06-02

**Authors:** Ramon Pires, Tiago Carvalho, Geoffrey Spurling, Siome Goldenstein, Jacques Wainer, Alan Luckie, Herbert F. Jelinek, Anderson Rocha

**Affiliations:** 1 Institute of Computing, University of Campinas, Campinas, São Paulo, Brazil; 2 The Southern Queensland Centre of Excellence in Aboriginal and Torres Strait Islander primary health care, Queensland Health, Brisbane, Australia; 3 Discipline for General Practice, School of Medicine, Brisbane, Queensland, Australia; 4 Retinal Division, Albury Eye Clinic, Albury, New South Wales, Australia; 5 Australian School of Advanced Medicine, Macquarie University, Sydney, New South Wales, Australia; 6 Centre for Research in Complex Systems and School of Community Health, Charles Sturt University, Albury, New South Wales, Australia; Bascom Palmer Eye Institute, University of Miami School of Medicine, UNITED STATES

## Abstract

Diabetic Retinopathy (DR) is a complication of *diabetes mellitus* that affects more than one-quarter of the population with diabetes, and can lead to blindness if not discovered in time. An automated screening enables the identification of patients who need further medical attention. This study aimed to classify retinal images of Aboriginal and Torres Strait Islander peoples utilizing an automated computer-based multi-lesion eye screening program for diabetic retinopathy. The multi-lesion classifier was trained on 1,014 images from the São Paulo Eye Hospital and tested on retinal images containing no DR-related lesion, single lesions, or multiple types of lesions from the Inala Aboriginal and Torres Strait Islander health care centre. The automated multi-lesion classifier has the potential to enhance the efficiency of clinical practice delivering diabetic retinopathy screening. Our program does not necessitate image samples for training from any specific ethnic group or population being assessed and is independent of image pre- or post-processing to identify retinal lesions. In this Aboriginal and Torres Strait Islander population, the program achieved 100% sensitivity and 88.9% specificity in identifying bright lesions, while detection of red lesions achieved a sensitivity of 67% and specificity of 95%. When both bright and red lesions were present, 100% sensitivity with 88.9% specificity was obtained. All results obtained with this automated screening program meet WHO standards for diabetic retinopathy screening.

## Introduction

Automated screening utilizing digital retinal cameras has been shown to achieve a similarly high level of accuracy to ophthalmologists, optometrists and trained general practitioners in addition to having the advantage of saving time by providing a quality control step, identification of DR-related lesions and finally an indication of whether referral for further retinal examination is required. Classical approaches for automated detection of diabetic retinopathy in clinical practice and research have concentrated on detection of only single lesions such as microaneurysms or hard exudates. In addition, the current generation of computer-based classifiers require training images from the same population that is being tested, preferably from the same retinal camera and with the same resolution in addition to specific pre-processing of images for each type of lesion and is therefore not ideal for multi-lesion detection [1–3] in diverse populations.

Automated computer detection of multiple lesions presented as single lesions or in combination of different lesions associated with DR from digital images has the potential to further improve access to primary care-based screening for Aboriginal and Torres Strait Islander peoples with diabetes by introducing an automated process for initial classification of images into those that require and those that do not require referral or further investigation [4]. This saves the time-consuming process of evaluating every image by specialists or trained primary care physicians. Using an automated classifier, specialists and primary care physicians can better use their time for patient consultation and reviewing images that have been identified abnormal by the automated screening process.

The methodology employed in this work for image representation is the Bag of Visual Words (BoVW), widely explored by the computer vision community [4–6]. This approach has an explicit and reformulated analogy with the traditional bag-of-words formalism, by associating the low-level local features to the elements of a visual dictionary. The visual dictionary, in general, is obtained by clustering algorithm or with a random selection. The association between local features and visual dictionaries produces a histogram named bag of visual words, that is used as a mid-level representation [5].

The objective of this study is to demonstrate that the BoVW-based automated diabetic retinopathy classification system can identify abnormal retinal images in an Aboriginal and Torres Strait Islander population.

## Methods

### Retinal Image Classification

One hundred and thirty two Aboriginal and Torres Strait Islander patients attending the Inala Indigenous Health Service, a Government primary health service in Brisbane, Queensland, for their annual diabetes cycle of care had retinal examinations as part of their diabetes health review between October 2007 and September 2009 [7]. These patients were largely female (55%) with a mean age of 52 (24–78 years) and 30% had some form of diabetic retinopathy. Two primary care physicians, who were accredited by the Royal Australian and New Zealand College of Ophthalmologists to read retinal photographs, interpreted the images and reviewed by the retinal ophthalmologist. Of the total 264 multi-color images available, thirty good quality retinal photographs with a range of diabetic retinopathy presentations and normal retinal images were selected as test images following permission from the patients to use the images for research. Ethics approval was obtained through the University of Queensland Human Ethics.

For this study, the retinal specialist (AL) reviewed all images and selected the current test images optimized for identification of bright and red lesions seen in diabetic retinopathy. Eighteen were marked as having no pathology, twelve were marked as red lesions (microaneurysms or haemorrhages), five contained bright lesions (hard exudates). The images that contain hard exudates also present signals of red lesions. The test images were captured with a Canon CR-DGi IOS 30D (Canon Australia Pty. Ltd. Sydney) non-mydriatic digital retinal camera by trained practice nurses after written informed consent, obtained from the patients. The camera settings were 15 × 22.5mm, 8.5 megapixels (Mp) and 45° field of view. All images were JPEG compressed at the minimal compression setting available as used in routine ophthalmological examination. Research adhered to the tenets of the Declaration of Helsinki. The images from the Inala Indigenous Health Service involve the use of images of Aboriginal and Torres Strait Islander peoples, a vulnerable group, and will require ethical oversight to be shared. Please contact Prof. Anderson Rocha (anderson.rocha@ic.unicamp.br) and Prof. Geoffrey Spurling (g.spurling@uq.edu.au) for an application to receive these images.

The training images for the visual word dictionaries came from the Ophthalmology Department, Federal University of São Paulo, Brazil, consisting of 687 non-DR images, 245 images with bright lesions and 191 images with red lesions (109 images contain signs of both bright and red lesions). The multi-color images came from a different ethnic group than Aboriginal and Torres Strait Islander patients (e.g., Caucasian) and were manually graded by three retinal specialists, and only the images for which the three specialists agree were kept in the final dataset. The images ranged from 640 × 480 to 1581 × 1113 pixels in resolution and were captured by a TRC50X (Topcon Inc., Tokyo, Japan) mydriatic camera and 45° field of view. These images, graded by the experts as containing or not containing specific lesions (inclusive multi-lesions), are available at http://dx.doi.org/10.6084/m9.figshare.953671.

The experiments were performed using the cross-dataset validation protocol, that consists of training the classifiers using a dataset and testing with another. The cross-dataset protocol is an important precaution in the design, since in clinical practice the images that need to be classified have rarely the same image specification (camera, resolution, operator, FOV) than the images used for training. Therefore, to highlight the effect of using, for training and testing, images from different populations, we also report results using the same dataset for training and testing. Therefore, we report results for both cases, (1) training with a diverse set of populations and ethnicity groups and testing with Aboriginal and Torres Strait Islander patients; and (2) training and testing, for showing calibration parameters, on non-Aboriginal and non-Torres Strait Islander patients (other ethnicities).

It is worth mentioning that the cross-dataset training and testing protocol is much more powerful than the traditional cross-validation protocol. In the cross-validation protocol, we obtain a set of images, normally with the same acquisition conditions and from the same population strata and train the algorithms. While testing, we will likely find testing examples following very similar conditions seen in the training. However, when using a cross-dataset protocol, it is unlikely to find, during testing, the same conditions. And this is what we show in this paper. We train our algorithms, for the first time in the literature, using a completely different population strata than the one found during testing. This is a remarkable achievement since populations such as Aboriginal and Torres Strait Islander patients are difficult to access and acquiring images of such groups for a proper training is very difficult and often not possible depending on the ethnic group. With a program like ours, the clinician can train the algorithms using any population strata for which he or she already has enough training examples and directly test remote-access populations such as the ones we deal with in this work, with no additional requirements.

### BoVW-based Representation

Images from the São Paulo Diabetes Hospital were used for creating the visual word dictionaries and for training the two-class classifiers, including the multi-lesion detector (normal vs. bright lesions and normal vs. red lesions as well as the multi-lesion vs. normal classifier). In this section, we present a brief introduction to the algorithm used in this paper for single-lesion and multi-lesion detection [8, 9].

Initially, regions within the retinal image that contain specific lesions are identified by ophthalmologists and, within these regions, the automated classification algorithm identifies Points of Interest (PoIs), which may be boundaries associated with the lesion or a color change.

**Fig 1:**
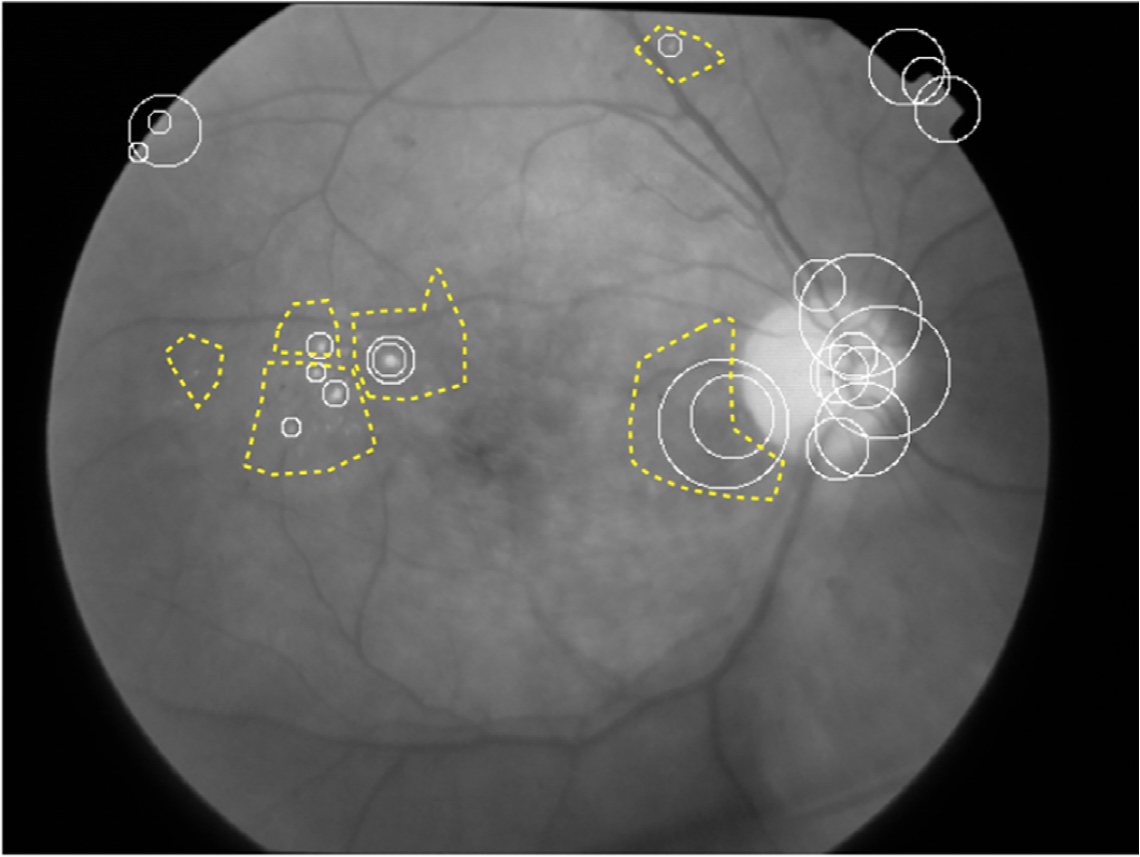
Retinal image. Regions of Interest (dashed line boundaries) and PoI (solid, circular boundaries).

A visual word, which represents the lesion characteristics, then consists of a group of similar PoIs within the region of interest (Fig 1) that are positive for the lesions of interest (e.g., microaneurysms and hard exudates). No specific regions of interest need to be identified within control images. The codebook learning can be performed by a k-means clustering over features chosen at random from a training set of images.

Besides the low-level descriptors (PoIs identification and description) and the codebook learning, two more fundamental steps are involved in the BoVW formalism: the coding and the pooling operations. The coding step transforms the PoIs into a representation based upon the visual words, which is better adapted to the specific task and preserves relevant information, while discarding noise. In this paper, we use the classical BoVW model, that employs the “hard assignment” of the PoIs to the closest visual word, associating each PoI fully and only to its closest visual word in the codebook. The classical pooling corresponds to a “counting of words” (called sum-pooling). The combination hard-sum coding/pooling is equivalent to a histogram that reveals the number of activations for each visual word.

An image can then be described by one or more visual words and the dictionary is constructed based on positive samples containing dot/blot hemorrhages and microaneurysms for red lesions and hard exudates for the bright lesions based on the identified discontinuities stored as visual words. Negative samples that contained no signs of disease are also included. The visual words dictionary then automatically classifies an image with unknown diabetic retinopathy.

The method was validated using the specificity and sensitivity result obtained from the receiver operating curves (ROC). The ROCs are calculated after training the classifier by stepwise changing the cut-off value, which determines whether a lesion is present or not. We also report the results in area under the curves (AUC) for comparison in the multi-lesion detection. The AUC gives a better overall performance measure than any particular point of the specificity-sensitivity metrics. We further considered a normalization technique applied to the raw data prior to classification of the lesion.

**Fig 2:**
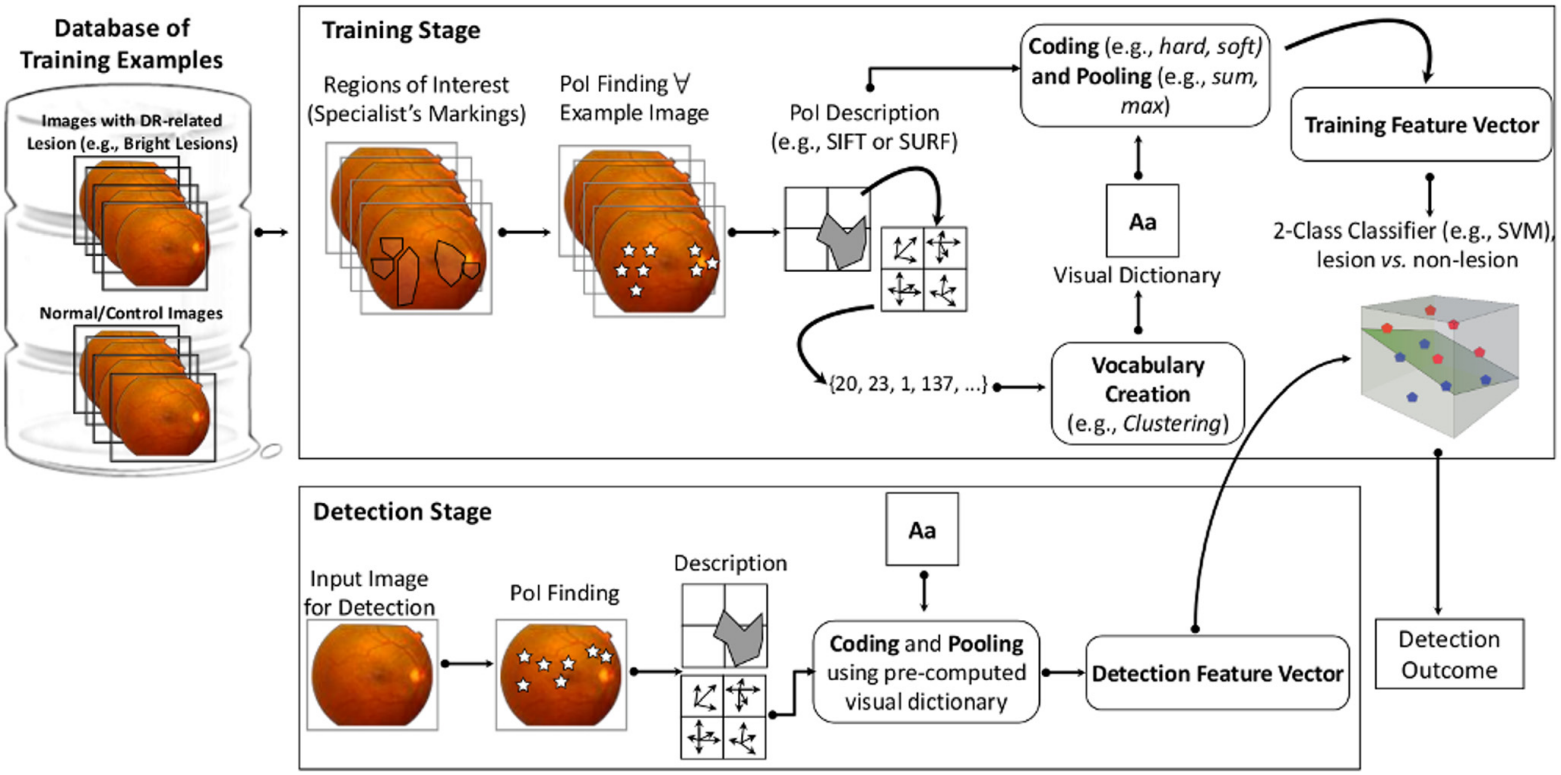
Pipeline of the BoVW-based automated diabetic retinopathy classification system for abnormal retinal images identification. For Low-Level feature extraction, the proposed method identifies Points of Interest (points in high-contrast or context-changing areas) within regions of interest which contain specific lesions marked and reviewed by specialists during the training of the method. For Codebook Learning (vocabulary creation), the method uses the k-means clustering method with Euclidean distance over a sample of the points of interest. The centroids are used as representatives codewords (the most important points of interests, for instance). With the codebook and the low-level description of a set of training images for a specific DR-related lesion, the Mid-Level feature extraction step employs the classical coding/pooling combination (hard/sum), that consists of defining a histogram that reveals the number of activations for each visual word in each analyzed image. For the Decision model training, the current method requires the training of one decision model for normal vs. bright lesions, normal vs. red lesions, or normal vs. multi-lesion classification.

Fig 2 illustrates the pipeline of the methodology of the BoVW-based system for identification of abnormal retinal images.

### Statistical analysis

A receiver operating curve (ROC) statistical analysis was applied to the data. The ROC is a “Graph of a function” plot which illustrates the accuracy of the classifier as the threshold for classification between two groups is varied. ROC plots the fraction of true positives out of the total actual positives giving the true positive rate (specificity) in relation to the percent of false positives (sensitivity) out of the total actual negatives. This study is an exploratory study to identify accuracy of the BoVW algorithm for detecting diabetic retinopathy lesions in Images of Aboriginal and Torres Strait Islander people where training images come from a different population and have a smaller resolution. In this work, we have 30 test images and 1123 training images.

## Results

Our experimental section has two main objectives. The first is to show that it is possible to obtain good DR-related lesion detection when training and testing using the same dataset, which means training and testing with images that come from similar population strata. This is not a novelty, but just a check whether the application is optimized and for using this as a reference in the second experiment where training is undertaken with images not from the same population strata as the test images.

The second objective is to verify if it is possible to train classifiers for DR lesion detection using images from a given population strata and test such classifiers with completely different images coming from another population strata. In this experiment we trained the classifier with a dataset collected in São Paulo, Brazil, comprising mostly Caucasian patients and testing the classifier on Aboriginal and Torres Strait Islander images, a completely different population strata. This is the most important part of this paper, as it compares how alike the training and testing outcomes are when using the same dataset and when different datasets are used. The results provide a very good indication that the bag-of-visual-words based detectors have the potential for generalization, at least for the population strata tested in this paper.

### Round #1: Training/Testing within a population strata

**Fig 3:**
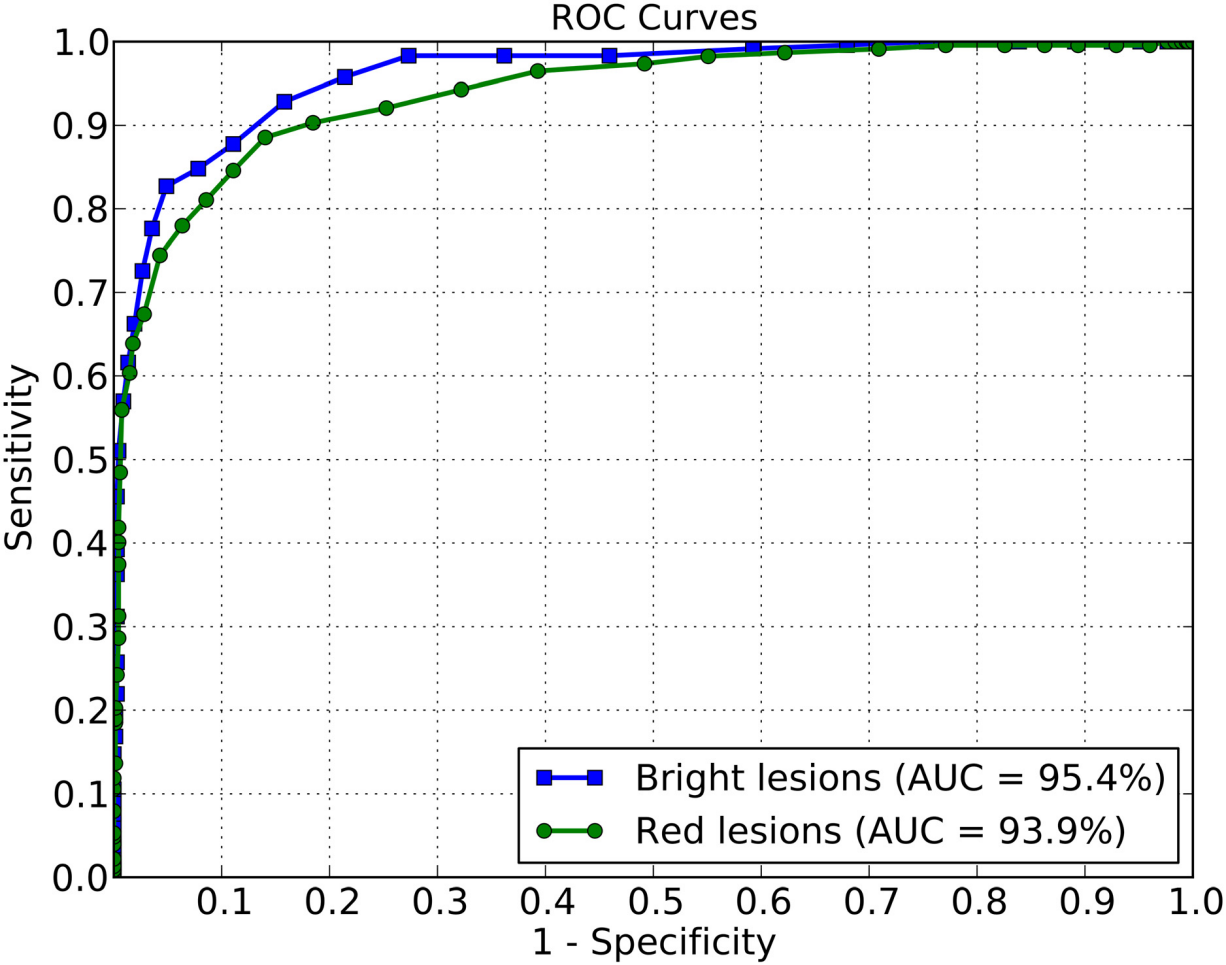
ROC curves. Training and testing images both from DR1 dataset.

To highlight the effect of using training images from a population that differs to test/screening images, we used the São Paulo retinal image database for both training and testing of diabetic retinopathy images. Fig 3 compares the ROC curves obtained when classification of images with unknown lesions was based on the training set being from the same population base. In this case, both the training and test set came from the São Paulo retinal image repository (DR1) dataset. For 90.0% specificity, the classifier achieved a sensitivity of 86.7% for bright lesions while for red lesions a sensitivity of 83.3% was obtained.

### Round #2: Training/Testing with different population strata

**Fig 4:**
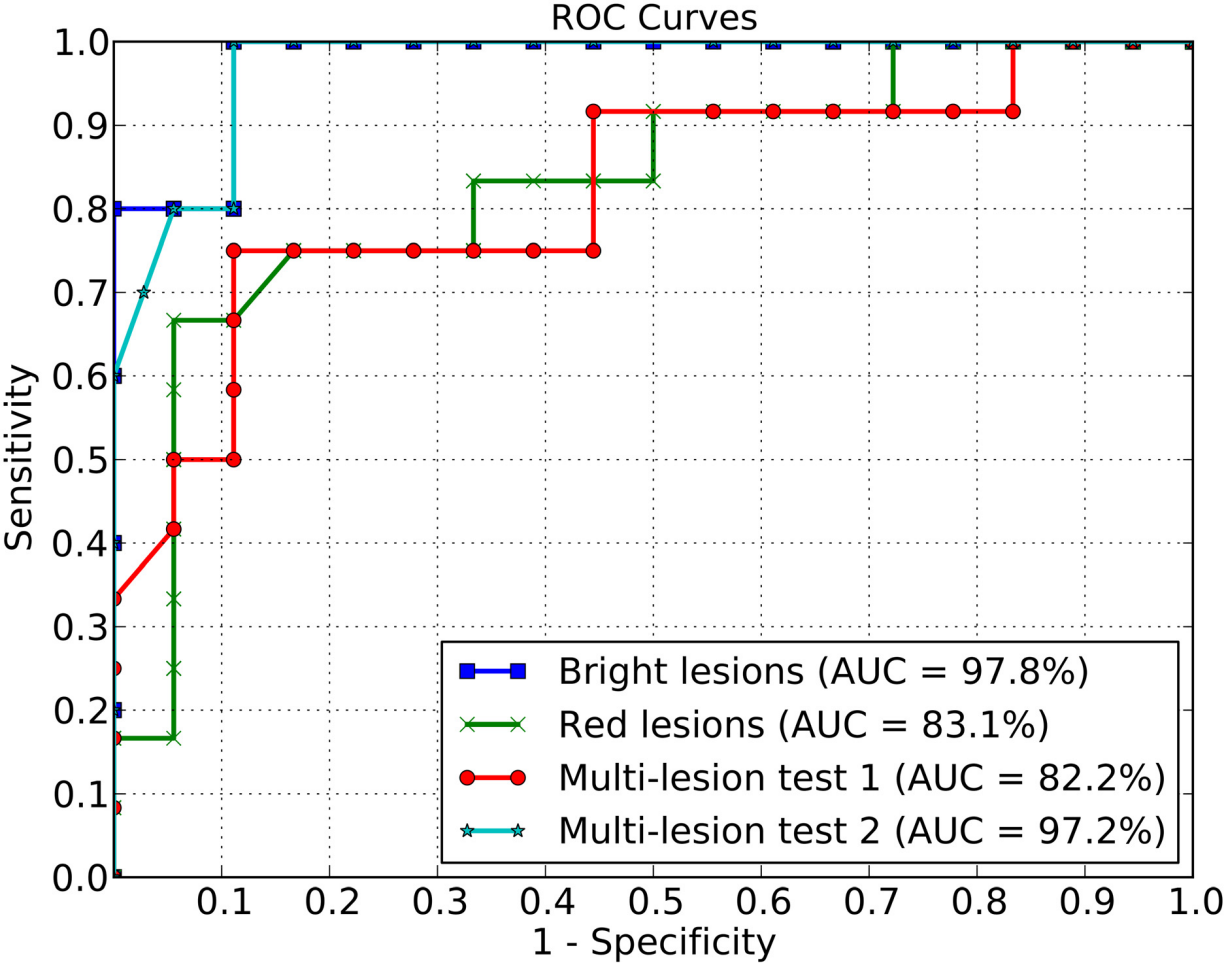
ROC curves. Red lesion, bright lesion, and multi-lesion detection.

We now turn our attention to showing that the developed lesion detectors have good generalisation power when training on one dataset and testing on another. In this case, we train the lesion detectors using the dataset collected in São Paulo, Brazil, DR1 dataset, which comprises mostly Caucasian peoples and test such classifiers with images from an Aboriginal and Torres Strait Islander population. The use of the visual word dictionary on this Aboriginal and Torres Strait Islander population sample achieved an AUC of 97.8%, with a sensitivity of 100.0% and specificity of 88.9% for bright lesion detection, as shown in Fig 4.

For red lesion detection, the classifiers achieved an AUC of 83.1% (Fig 4), with 67.0% sensitivity and 95.0% specificity.

The multi-lesion classifier combined the red and white lesion classifier outcomes. The image is classified as normal if both the red and bright lesion classifiers agree it is normal, which minimizes the classification of false-negatives.

For identification of multiple lesions, two different test sets were chosen. The first set contained a mix of 18 normal images and 12 images with red, bright, or both types of lesions (5 images), representing what may be seen in a screening program. An accuracy of 82.2% was achieved with a sensitivity of 75.0% and specificity of 88.9%. The ROC curve is represented in Fig 4 as “Multi-lesion test 1”.

The second test compared 18 normal images and 5 images with both lesions (red and bright lesions) present. The result achieved an accuracy of 97.2%, associated with a sensitivity of 100.0% and specificity of 88.9%. The ROC curve is represented in Fig 4 as “Multi-lesion test 2”.

#### Comparison with the State of the Art

For any automated computer-based screening to be clinically useful, it needs to meet standards such as the St Vincent declaration, which recommends a sensitivity of 80% and specificity of 95% in agreement with the British Diabetic Association. The Australian National Health and Medical Research Council (NHMRC) guidelines for primary health care require 60% sensitivity with 90–95% specificity [10–12]. The current study investigated a multi-lesion classifier that does not require pre- or post-processing, and is robust against differences between training and testing images as well as differences in ethnicity and meets the NHMRC standards.

Our result for determining red lesions in Aboriginal and Torres Strait Islander images outperforms previously reported results within the acceptable range for Australian screening programs [12], although the test image battery was smaller compared to previous reported studies and the test images were of higher resolution than the training images. In addition, the number of training images with only red lesions was smaller than the training set for bright lesions.

Bright lesion detection for diabetic retinopathy using the visual word dictionary was highly accurate being equal to or improving on previous studies. Our results indicated 100% sensitivity and specificity of 88.9% despite the use of a generic training set which included no Indigenous images nor pre- or post-processing of any kind. By comparison, a recent study by Niemeijer et al. achieved a sensitivity of 95.0% and specificity of 88.0%, for the detection of bright lesions [13], albeit in a non-Aboriginal cohort.

Ideally, automated multi-lesion detection systems should be able to detect specific lesions present in any combination and at any time of retinopathy progression. Only a few studies have reported on multi-lesion detection in diabetic retinopathy. Abràmoff et al. combined existing software from diverse laboratories and achieved a sensitivity of 84.0% with 64.0% specificity (accuracy 87.0%) [14]. A larger cohort improved the accuracy to 90.0%. Fleming et al. reported that inclusion of exudate and blot haemorrhages detection improved the overall sensitivity of detecting referable retinopathy from 94.9% to 96.6% [1]. The exudate detection algorithm proposed by Fleming and collaborators is based on the microaneurysm detector previously reported and adds white lesion detection [15]. Separate pre-processing of images with white and red lesions being detected in sequential steps as well as requiring prior identification and removal of poor quality images is required by this software. A more recent study by Abràmoff et al. also discussed referable retinopathy accuracy but did not discuss bright or red lesion detection accuracy nor multi-lesion detection [16].

## Discussion

Diabetic retinopathy (DR) is present in approximately 25.4% of people with diabetes. In Australia, it affects up to 30% of some Aboriginal and Torres Strait Islander communities [17]. Despite a higher prevalence of DR, Aboriginal and Torres Strait Islander people experience fewer retinopathy screening opportunities owing to difficulties in accessing optometry or ophthalmology services [18]. To improve eye health and address diabetic retinopathy specifically, the Inala Indigenous Health Service (IIHS) introduced a regular clinic-based digital retinal photographic screening service. The use of a digital retinal camera located in a culturally safe primary health care service improved access to diabetic retinopathy screening six-fold for Aboriginal and Torres Strait Islander clients [7]. The use of one or two fields of view from the retinal fundus (usually the posterior pole and the optic disc centred) as in the Inala study has been shown to be as effective as the traditional seven-field ETDRS requirement [19]. At the IIHS, retinal photos are read by specially trained primary care physicians and images stored on site. The current study discusses the ability of computerized image analysis software to detect diabetic retinopathy with microaneurisms, dot/blot hemorrhages, hard exudates, or multiple lesions from a clinical standpoint using a single algorithm. While the software does require “training” on known images, the advantage of the Bag of Visual Words software is that it can be trained on images from other populations, cameras, and resolution. Also, this software does not require any special pre-processing or screening of images.

For both bright and red lesions, Figs 3 and 4 indicate that, for a 90.0% specificity, the sensitivities reached using the DR1 for training and testing dataset are superior than the sensitivities obtained for the cross-dataset protocol. However, the AUC gives a better overall performance measure than any particular point of the specificity-sensitivity metrics. The AUC achieved for bright lesions when training/testing on different populations surpasses the AUC obtained for training and testing with DR1.

A possible explanation for this difference in results between cross-dataset validation (training with DR1 and testing with different populations) and cross-validation protocol (training and testing with DR1, mostly the same population groups in training and testing), is that the patients present in the Aboriginal and Torres Strait Islander populations in our dataset presented lesion signs that were more visibly detectable than the lesions contained in the images of the training data in DR1 dataset (images from São Paulo). When that happens, the testing conditions are slightly better behaved as they provide more details to the algorithm.

The visual word dictionary classifier is a novel program for multi-lesion detection, which obtained a 100.0% sensitivity with 88.9% specificity for lesion identification if bright and red lesions were both present in the same image. The automated multi-lesion diabetic retinopathy detection program proposed here identifies white and red lesions following training of the classifier with images of known retinopathy [20, 21]. The advantages of the visual words method are that it does not require pre- or post-processing of images as is currently the case, and allows the classifier to be trained on images not from the current clinical population.

The limitation of the study was the number of retinal images obtained from the Inala Health Care Centre. The images used for testing were selected to show proof of concept that the visual words dictionary classifier is able to detect white and red lesions regardless of whether single lesions (microaneurysms or hard exudates) are present or if they are mixed in a retinal image.

To confirm these findings, we will apply the visual words classifier to a larger more clinically representative sample of images where the population incidence of retinopathy is considered and reflected in the percentage of control and retinopathy images included in the training and test sets. It is worth mentioning that number of enough-quality retinal images obtained from the Inala Health Care Centre of the study is not large. However, we would emphasize that it can be difficult to develop a mutually beneficial research process based on trusting relationships with Indigenous populations which is a necessary prerequisite to the use of images and medical data. In addition, although the study is still preliminary, and a larger study with more patients and images would be interesting, there is already a good indication of the generalization capability of this kind of classifiers for this problem.

Diabetic retinopathy screening has concentrated on identification of specific single lesion types and the location of these lesions within the retina. Classifiers have had to be specific for each lesion and trained from a set of images from the same population and resolution, preferably with the same camera. Although high accuracy has been achieved, there may not be a need to identify the location of the lesion at screening in the first instance. The important issue is whether diabetic retinopathy signs are present and whether a referral for further review by a specialist is required. Regular assessment for diabetic eye disease is not only difficult in rural and remote areas and developing countries, but also in urban areas where functional access to diabetic retinopathy screening may be low for cultural reasons. The experience at Inala has shown that urban Indigenous people may have very limited access to mainstream services like the ophthalmology outpatients departments of big urban hospitals [7]. Methods that simplify the screening process, increase efficiency, and reduce the costs of retinal screening have the potential to confer significant health benefits [22].

Digital image acquisition and computer-based assessment provides the opportunity for effective, on-the-spot screening by non-specialists using automated procedures and also effective transfer of images and results to central health care agencies and specialists [23, 24]. Many programs are now available or being developed for detection of diabetic retinopathy [15, 25–27]. However, each focuses on a different set of lesions, which have been tested in ideal laboratory conditions and often involve different computer pre- and post-processing requirements. These requirements are not easily combined and implemented in screening programs for detection of multiple lesions. A major advantage of automated identification using the visual word library is that any lesion or multiple types of lesions in the same image can be accurately identified even in images of different ethnicity and analyzed at different levels of resolution [20, 21].

The visual words multi-lesion classifier is a more accurate system based on categorizing when both lesions are present in the retinal images (100.0% sensitivity and 88.9% specificity), which also complies with WHO and Australian NHMRC guidelines [3, 12, 14].

The main contribution of this paper is the description and the use of a cross-training methodology with results obtained from training with images from a particular ethnic group, and testing with a different population. This has very important health screening and treatment impacts as the program can be trained at metropolitan clinics and taken to rural and remote areas for live screening and have a high degree of accuracy. The importance of this approach is that training images are not always available from remote communities in enough numbers to train the classifier and many small towns consist of a very heterogeneous ethnic population mix.

Future work will have to address affordability, acceptance and accuracy in a larger representative cohort study. We conclude that the current results indicate that the visual word dictionary is unique as it is able to accurately indicate the presence of either white or red lesions as well as multi-lesions in retinal images with diabetic retinopathy of different resolution and from diverse ethnic backgrounds.
